# An enhanced fruit fly optimization algorithm with random spare and double adaptive weight strategies for oil and gas production optimization

**DOI:** 10.1038/s41598-025-15205-3

**Published:** 2025-08-09

**Authors:** Xu Wang, Jingfu Shan

**Affiliations:** 1 School of Geosciences, Yangtze University, Caidian, Wuhan, 430100 China; 2 Key Laboratory of Exploration Technologies for Oil and Gas Resources, MOE, Yangtze University, Wuhan, 430100 China

**Keywords:** Fruit fly optimization algorithm, Parameter optimization, Global optimization, Oil and gas production optimization, Energy science and technology, Engineering, Mathematics and computing

## Abstract

In the field of petroleum extraction, enhancing oil and gas recovery processes is essential for sustaining the economic viability of energy enterprises and addressing the continuously increasing global energy demand. Efficient subsurface production plays a pivotal role in strategic decision-making, including the selection of optimal drilling sites and the determination of effective well control parameters. However, conventional reservoir optimization techniques are often computationally intensive and may struggle to deliver satisfactory solutions. As a promising alternative, evolutionary algorithms—rooted in the principles of natural selection—have demonstrated strong potential for addressing complex optimization problems due to their gradient-free nature and inherent suitability for parallel computation. In this study, we propose an enhanced evolutionary algorithm tailored for global optimization and oil and gas production improvement. This method builds upon the original Fruit Fly Optimization Algorithm (FOA) by incorporating a random spare mechanism and a dual adaptive weighting scheme, aiming to achieve a more effective balance between exploration and exploitation during the search process. Specifically, after the standard FOA updates the population, the random spare mechanism is introduced to enhance exploratory capabilities and avoid premature convergence. Subsequently, the dual adaptive weighting strategy is employed to improve convergence speed and solution refinement. The proposed RDFOA algorithm is rigorously validated through comprehensive experiments on benchmark test suites from IEEE CEC 2017 and IEEE CEC 2022. These evaluations include ablation studies, scalability analyses, visualization of search trajectories, and comparative assessments against state-of-the-art algorithms. On the CEC 2017 benchmark, RDFOA outperforms CLACO in 17 functions and QCSCA in 19 functions. On the CEC 2022 benchmark, it surpasses CCMSCSA and HGWO in 10 functions. The experimental results clearly demonstrate that RDFOA consistently achieves superior performance in oil and gas production optimization scenarios.

## Introduction

In the petroleum sector, reservoir management (RM) remains a crucial area of interest for oil and gas enterprises. As highlighted by Wiggins and Startzman^1^, RM entails the deliberate coordination of technological tools, financial resources, and human capital to improve both the economic viability and recovery efficiency of hydrocarbon reservoirs. They further emphasized that reservoir management (RM) encompasses the entire life cycle of a reservoir—from initial discovery to final decommissioning. Within this framework, production optimization constitutes a central function of RM. Oil and gas producers continually seek to enhance hydrocarbon output, not only to meet the escalating global energy demand but also to maximize economic returns.

One widely employed technique for enhancing oil production is water injection, commonly known as waterflooding. This method enables the recovery of additional hydrocarbons beyond what is achievable through natural drive mechanisms, such as gas cap expansion and gravity segregation^2^. However, the effective implementation of waterflooding demands meticulous planning to avoid cost inefficiencies and operational challenges. As a result, the optimization of waterflooding strategies has long been a central focus in both industry and academia, aimed at enhancing recovery performance and overall cost-effectiveness.

The task of waterflooding optimization poses a complex engineering problem, often addressed using mathematical models and algorithms to fine-tune design parameters^3^. These parameters can include production rates, bottomhole pressures, and the scheduling of water injection processes. In more advanced scenarios, the problem is formulated as a multi-objective optimization task, where competing goals are balanced simultaneously^4,5^. This formulation reflects the multifaceted nature of real-world reservoir management and offers engineers practical decision-making insights.

A critical tool in this context is numerical reservoir simulation (NRS), which is widely employed during the reservoir development phase to model subsurface fluid flow dynamics. When coupled with optimization techniques, NRS plays a pivotal role in refining production strategies. However, a significant limitation of NRS is its high computational cost, particularly when simulating geologically complex reservoirs. As NRS is grounded in physical and mathematical formulations of fluid behavior, increased geological heterogeneity directly translates into longer simulation times. Consequently, mitigating this computational burden has emerged as a key focus of ongoing research.

Classic global optimization problems are often based on gradient information, containing a large amount of local optimal solution disturbance information and nonlinear obstacles^6^. In recent decades, researchers have proposed many metaheuristic methods to overcome these difficulties^7–10^. These methods are very simple, inspired by various natural phenomena, with efficient calculation methods and inexpensive computational costs. Notable among them are: the firefly algorithm (FA)^11^, differential evolution (DE)^12^, bat algorithm (BA)^13^, multi-verse optimization algorithm (MVO)^14^, dragonfly algorithm (DA)^15^, Gyro Fireworks Algorithm^16^, Frigatebird Optimizer^17^, Fishing Cat Optimizer^18^, grey wolf optimization (GWO)^19^. The fruit fly optimization algorithm (FOA) is a newer swarm intelligence algorithm that simulates the foraging behavior of fruit flies using visual and olfactory mechanisms. Nevertheless, the no free lunch (NFL) theorem asserts that no optimizer can be universally optimal for all types of problems^20^. As a result, a wide range of evolutionary and swarm-based optimization algorithms have been proposed in the literature. An ideal optimization algorithm would yield a near-optimal solution tailored to the specific problem at hand. However, the “No Free Lunch” theorem underscores that no single optimization method performs best across all problem domains. An algorithm that excels in one class of optimization problems may underperform in another, potentially converging prematurely to local optima or failing to find satisfactory solutions. While population-based algorithms often demonstrate promising results in continuous optimization tasks, they may struggle with discrete problems such as feature selection. Additionally, many population-based methods suffer from instability, with performance varying considerably across runs. These methods often face challenges in maintaining an effective balance between exploration and exploitation during the search process.

Many researchers are studying the issue of oil and gas extraction, among which evolutionary algorithms are an effective method to deal with the problem of oil and gas extraction. for example, Wang et al.^21^ proposed an evolutionary algorithm-based framework for addressing oil and gas production optimization problems under uncertain operational conditions. Their approach enables real-time and robust decision-making by formulating the optimization process as a Markov decision chain. Within this framework, reinforcement learning (RL) techniques are employed to train and iteratively refine control strategies aimed at maximizing production objectives. To enhance the learning efficiency and adaptability of RL agents, the method integrates evolutionary algorithms into the training process. The evolutionary component strengthens the convergence behavior of the RL models, improves the agents’ decision-making capabilities, and accelerates convergence toward optimal control policies. Du et al.^22^ proposed an optimization framework based on evolutionary algorithms, which integrates surrogate methods, uses Bayesian random forest techniques, and employs particle swarm optimization. The Bayesian random forest technique constructs a surrogate model that determines the working mechanism of the injection production system. This model can efficiently predict a parameter based on real-time data from injection and production, which determines the dynamic production parameters of the production well. This method utilizes particle swarm optimization algorithm to optimize the injection strategy and obtain an optimal solution, which can lead to the optimal injection strategy. Ng et al.^23^ also used particle swarm optimization algorithm to optimize production. Differently, this method utilizes long short-term memory algorithms. Long short-term memory method is a machine learning approach mainly used in the field of natural language processing. Particle swarm optimization algorithm is integrated with long short-term memory algorithm, and a large number of real cases are used to improve the accuracy of the algorithm. Other related studies are summarized in Table 1.

**Table 1:** Related studies.

Study	Publication years	Main contributions	Strengths	Weaknesses
Wang et al.^21^	2023	Combined reinforcement learning with evolutionary algorithms for robust decision-making under uncertainty.	Enhances convergence and decision-making of RL; suitable for uncertain environments.	Requires significant training time; performance depends on RL settings.
Du et al.^24^	2025	Combined genetic PSO with policy gradient reinforcement learning to adaptively adjust parameters.	Avoids manual tuning; adaptive parameter control; effective in complex scenarios like hydraulic fracturing; improved performance over GAPSO.	Higher runtime due to RL component (~ 7.3%); performance depends on policy network design.
Du et al.^22^	2023	Proposed a surrogate-assisted framework using Bayesian random forest + PSO.	Efficient prediction using surrogate model; effective injection strategy optimization.	Surrogate modeling may reduce accuracy in highly dynamic systems.
Ng et al.^23^	2023	Integrated PSO with LSTM for production optimization using real data.	High accuracy due to LSTM; effective in modeling time-dependent production behavior.	LSTM model training is complex; risk of overfitting on small datasets.
Shi et al.^25^	2025	Built multiple intelligent models for drilling rate prediction and optimized them via GA.	GA-LGBM achieved highest accuracy and robustness; interpretable via SHAP; validated on field data.	Some models (e.g., KNN, MLP) may underperform in noisy datasets.

Although substantial research has been conducted within the academic community, existing algorithms still exhibit notable limitations in balancing exploration and exploitation, often failing to consistently yield globally optimal solutions. According to the No Free Lunch (NFL) Theorem, algorithmic performance is inherently problem-dependent; thus, tailoring existing algorithmic frameworks to specific problem types can help overcome their fundamental bottlenecks and lead to the development of more robust variants. Guided by this principle, the present study introduces targeted enhancements to the classical Fruit Fly Optimization Algorithm (FOA), aiming to improve its performance stability and computational efficiency across complex optimization tasks.

In summary, the main contributions of this study are outlined as follows:

1. This study introduces an enhanced version of FOA, referred to as RDFOA, which incorporates the random spare strategy and double adaptive weight strategy.
2. This study systematically verified the comprehensive advantages of RDFOA in convergence, robustness, and dimensional scalability through rigorous comparative experiments and mechanism decoupling analysis.
3. To evaluate the performance of the proposed RDFOA in real-world production challenges, this study applies it to optimize production in three-channel reservoirs. The experimental findings emphasize the outstanding optimization capabilities of the proposed algorithm in practical settings.

This paper is partially structured as follows: “Fruit fly optimization algorithm” provides a comprehensive background on the FOA. Section “Proposed RDFOA” elaborates on the two new mechanisms integrated into the FOA. In “Experiments and results”, a series of comparative experiments are conducted to evaluate the RDFOA. Section “Application to oil reservoir production” summarizes the chapter and suggests directions for future research.

## Fruit fly optimization algorithm

**Fig. 1:**
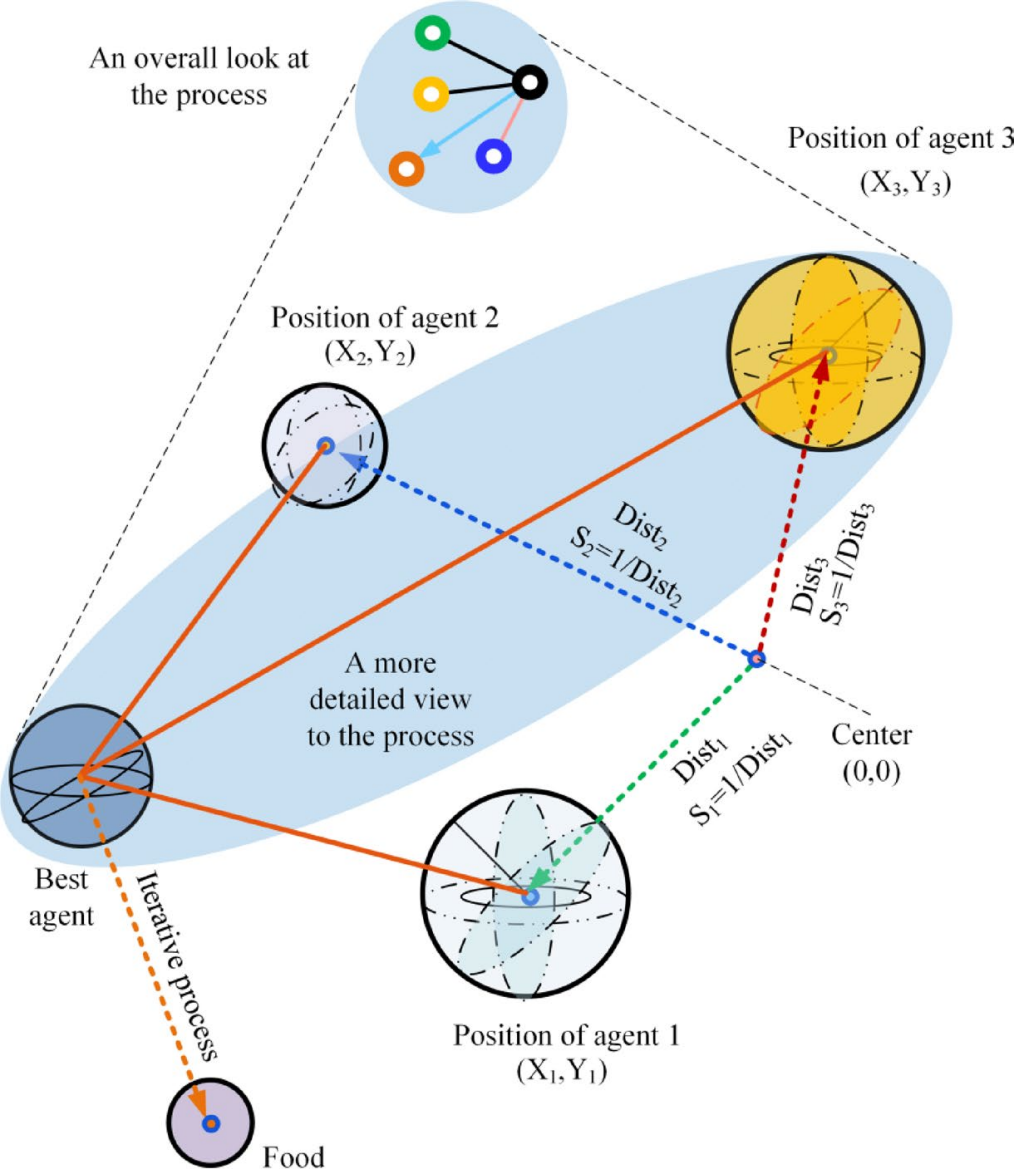
Iterative foraging process of fruit flies.

In 2012, Pan introduced the Fruit Fly Optimization Algorithm (FOA)^26^. The Fruit Fly Optimization Algorithm (FOA) is a bio-inspired optimization technique that simulates the foraging behavior of fruit flies. FOA leverages the olfactory and visual perception capabilities of fruit flies to identify optimal solutions within a defined search space. During the initial phase of the search, individuals move in random directions guided by their acute sense of smell, traversing varying distances to explore potential food sources. As they approach a food source, their behavior shifts from smell-driven exploration to vision-based localization to enhance accuracy. Once a promising location is identified and evaluated, the flies assess its quality and disseminate this information to the swarm. Subsequently, the population converges toward the most favorable region. From this new base, the search continues in an iterative manner to discover even better solutions. The overall exploratory behavior of FOA is depicted in Fig. 1.

The execution process of FOA algorithm includes the following steps:

The first step is the parameter initialization phase. At this stage, it is necessary to set the maximum number of evaluations (MaxFEs), population size (popsize), and location range (LR). Subsequently, the initial positions of fruit fly individuals are randomly generated within the defined area to form the initial population.

1 $$\:{X}_{axis}=\text{r}\text{a}\text{n}\text{d}\left(\text{L}\text{R}\right)$$

2 $$\:{Y}_{axis}=\text{r}\text{a}\text{n}\text{d}\left(\text{L}\text{R}\right)$$

Step 2 Fruit flies use their olfactory perception mechanism to randomly conduct preliminary searches for food sources.

3 $$\:{X}_{i}={X}_{axis}+tempx$$

4 $$\:{Y}_{i}={Y}_{axis}+tempy$$

5 $$\:tempx=rand\:in\:\left[-\text{10,10}\right]$$

6 $$\:tempy=rand\:in\:\left[-\text{10,10}\right]$$

Step 3 Firstly, calculate the distance between each individual and the initial reference point using a specific formula;

7 $$\:{Dist}_{i}=\sqrt{{X}_{i}^{2}+{Y}_{i}^{2}}$$

Then, the reciprocal of this distance is used as an estimate of the individual’s perceived odor concentration.

8 $$\:{S}_{i}=1/{Dist}_{i}$$

Step 4 Substitute into the concentration evaluation function to calculate the olfactory fitness of each fruit fly’s current position.

9 $$\:{Smell}_{i}=Fitness\:function\left({S}_{i}\right)$$

Step 5 Identify the fruit fly with the highest odor concentration among all individuals as the current optimal solution.

10 $$\:\left[bestSmell\:bestindex\right]=\text{m}\text{i}\text{n}\left(Smell\right)$$

Step 6 The algorithm retains the optimal odor concentration value of the fruit fly and its position coordinates determined through visual perception, in order to guide the next round of search behavior.

11 $$\:{X}_{axis}=X\left(bestindex\right)$$

12 $$\:{Y}_{axis}=Y\left(bestindex\right)$$

13 $$\:Smellbest=bestSmell$$

Step 7 Iteration process. If the current odor concentration value is lower than the odor concentration value of the previous generation, repeat steps 2–5. Otherwise, proceed to step 6.

The implementation of the Fruit Fly Optimization Algorithm (FOA) can be outlined as follows.

**Figure:**
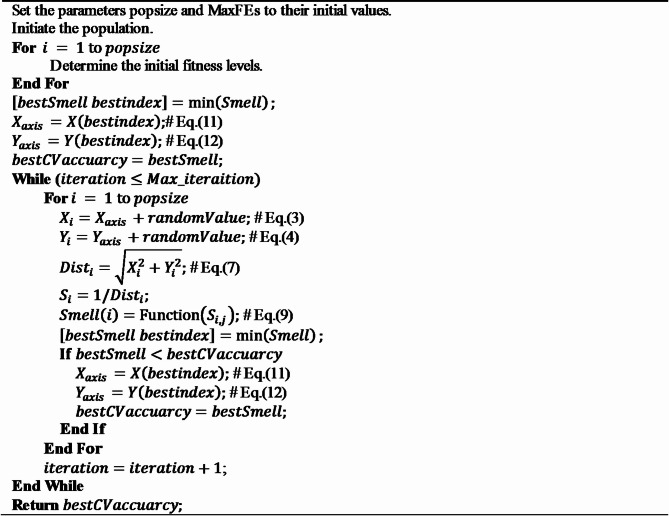
**Algorithm 1** FOA.

## Proposed RDFOA

The original Fruit Fly Optimization Algorithm (FOA) is recognized for its simplicity, minimal control parameters, and ease of implementation. However, despite these advantages, its performance remains suboptimal when addressing multi-modal and complex optimization problems, particularly in terms of convergence speed and solution accuracy. To address these limitations, this study proposes two enhancement mechanisms designed to improve both the convergence rate and solution quality of the classical FOA.

### Random spare strategy

The random spare mechanism operates by replacing the value of the current individual’s position vector at the *n*-th dimension with the corresponding component from the best-performing individual. In evolutionary search, it is common for certain dimensions of a candidate solution to be well-optimized while others remain suboptimal. In contrast, the global best individual typically demonstrates more consistent performance across all dimensions. To address this imbalance, we introduce a probabilistic, dimension-wise random replacement strategy that is applied from a starting dimension *m* through to the end of the vector. Empirical analysis indicates that setting *m* = 0 yields the most favorable results, suggesting that applying the strategy across the entire vector enhances performance. The decision to execute this replacement is governed dynamically by comparing a Cauchy-distributed random number with the ratio of the current evaluation count to the total number of evaluations, ensuring adaptivity throughout the optimization process.

The formula for the random spare mechanism can be expressed as Eq. (14).

14 $$\:{x}_{i,j}=\left\{\begin{array}{c}{x}_{\text{b}\text{e}\text{s}\text{t},j},\hspace{0.25em}\hspace{0.25em}\hspace{0.25em}\hspace{0.25em}if\:j\ge\:m\:and\:r<\frac{{F}_{\text{c}\text{u}\text{r}}}{{F}_{\text{m}\text{a}\text{x}}}\\\:{x}_{i,j},\hspace{0.25em}\hspace{0.25em}\hspace{0.25em}\hspace{0.25em}otherwise\hspace{0.25em}\hspace{0.25em}\hspace{0.25em}\hspace{0.25em}\end{array}\right.$$

Among them, $\:{F}_{\text{c}\text{u}\text{r}}$ represents the current number of evaluations, and $\:{F}_{\text{m}\text{a}\text{x}}$ represents the maximum number of evaluations. *r* represents a random number sampled from the standard Cauchy distribution, with *m* as the starting dimension index and empirically set to 0.

### Double adaptive weight strategy

Weight plays a crucial role in PSO, and various studies have explored adaptive weight adjustments to enhance the algorithm’s performance. Drawing inspiration from PSO, this study introduces dual adaptive weights to modulate the local and global search capabilities of the algorithm. In scenarios involving multi-peak functions, the original algorithm tends to converge prematurely to local optima. To mitigate this, we introduce weight $\:{w}_{1}$ to enhance global search and weight $\:{w}_{2}$ to bolster local search capabilities in later stages. These weights, formulated in Eq. (15) and Eq. (16), dynamically adjust during the optimization process.

15 $$\:{w}_{1}={(1-FEs/MaxFEs)}^{1-\text{t}\text{a}\text{n}(pi\times\:\left(rand-0.5\right)\times\:s/MaxFEs)}$$

16 $$\:{w}_{2}={(2-2\times\:FEs/MaxFEs)}^{1-\text{t}\text{a}\text{n}(pi\times\:\left(rand-0.5\right)\times\:s/MaxFEs)}$$

The parameter s adjusts according to the local optima encountered by the algorithm. Initially, s increments automatically when individual positions remain unchanged; subsequent updates halve s to moderate its impact. Due to the incorporation of s and Cauchy random numbers, weights $\:{w}_{1}$ and $\:{w}_{2}$ do not diminish linearly but fluctuate, especially as the algorithm confronts local optima. FEs denotes the current evaluation count, incremented with each evaluation, up to the maximum value of MaxFEs set at 300,000. The range for $\:{w}_{1}$ spans [0, 1], while that for $\:{w}_{2}$ spans [0.5, 1]. Notably, $\:{w}_{1}$ affects the first half of the algorithm, transforming equations such as into Eqs. (17), (18), and (19).

17 $$\:X\left(t+1\right)={w}_{1}\times\:{\text{X}}^{*}\left(\text{t}\right)-A\times\:D$$

18 $$\:X\left(\text{t}+1\right)={w}_{1}\times\:{\text{X}}^{*}\left(\text{t}\right)+{\text{D}}_{p}{e}^{bl}\text{c}\text{o}\text{s}\left(2\pi\:l\right)$$

19 $$\:X\left(t+1\right)={{w}_{1}\times\:\text{X}}_{rand}-A\times\:D$$

In the latter stage of the algorithm, weight $\:{w}_{2}$ is introduced, resulting in transformations such as Eqs. (20), (21), and (22).

20 $$\:X\left(t+1\right)={X}^{*}\left(t\right)-{w}_{2}\times\:A\times\:D$$

21 $$\:X\left(\text{t}+1\right)={X}^{*}\left(t\right)+{w}_{2}\times\:{D}_{p}{e}^{bl}cos\left(2\pi\:l\right)$$

22 $$\:X\left(t+1\right)={X}_{rand}-{w}_{2}\times\:A\cdot D$$

### The proposed RDFOA

In this section, we integrate the two mechanisms into the traditional FOA and provide a detailed description of the complete RDFOA process. The RDFOA starts by utilizing a random spare mechanism to improve the efficiency of finding global optimal solutions. Concurrently uses the double adaptive weight mechanism to expedite the algorithm’s convergence.

The pseudo-code of RDFOA is presented in Algorithm 2. RDFOA is an advanced metaheuristic algorithm inspired by the foraging behavior of fruit flies, designed to address complex optimization problems through iterative, population-based search strategies. The algorithm begins by initializing essential parameters and generating an initial population of fruit flies, each assigned random coordinates along the X and Y axes within the defined search space. For each individual, fitness values are calculated using a predefined fitness function. The fruit fly exhibiting the minimum fitness value, denoted as *bestSmell*, is identified, and its corresponding index (bestIndex) is recorded. The coordinates of this best-performing individual are then set as the reference positions (X_axis and Y_axis), and its fitness value is stored as bestCVaccuracy.

The main loop continues to execute as long as the current iteration count remains below the predefined maximum iteration limit (Max_iterations). In each iteration, the position of each fruit fly is updated by adding random offsets to the reference X and Y coordinates, simulating the exploratory search behavior. The updated positions are then evaluated using the fitness function to determine the fitness value at each location. During this process, the fruit fly with the lowest fitness value—referred to as the current bestSmell—is identified. If this fitness value surpasses the previously recorded optimal cross-validation accuracy (bestCVaccuracy), the corresponding X and Y coordinates are adopted as the new optimal position, and bestCVaccuracy is updated accordingly.

To further enhance the optimization, sensory values are refined through a random spare mechanism, which introduces diversity by probabilistically replacing certain positions, and a dual adaptive weighting mechanism, which dynamically adjusts the influence of different positions to balance exploration and exploitation. After updating, the iteration counter increments and the process repeats. Upon termination, the algorithm returns the best fitness value found, bestCVaccuracy, representing the optimal solution.

This systematic approach ensures that RDFOA effectively explores the search space and converges toward high-quality solutions, thereby demonstrating robustness and efficiency across a broad spectrum of optimization problems.

The time complexity of the RDFOA algorithm is mainly determined by its core computational operations. While retaining the original FOA algorithm’s O (T × N × D) basic complexity (T is the number of iterations, N is the population size, and D is the problem dimension), the introduction of random spare mechanism and double adaptive weight mechanism brings controllable additional computational overhead. The time complexity of random spare mechanism is O (N × D), and the time complexity of double adaptive weight mechanism is O (T × N × D), these improvements only increase the total complexity to the same order of O (T × N × D).

**Figure:**
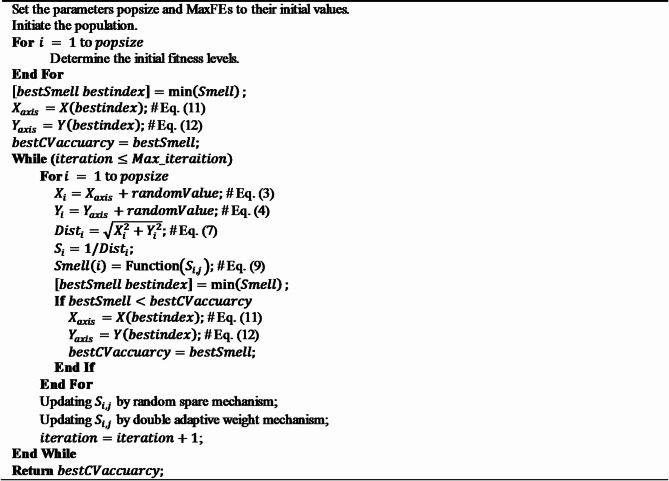
**Algorithm 2** Pseudo-code of RDFOA.

## Experiments and results

This section provides a comprehensive evaluation of the proposed algorithm through a series of rigorous experiments. The assessment encompasses ablation studies, scalability and extension tests, analysis of search trajectory histories, comparative performance evaluations against established algorithms, and the application of the developed COFOA to real-world engineering problems.

### Benchmark functions

#### IEEE CEC 2017 benchmark functions

Table 2 shows the details of the IEEE CEC 2017 benchmark functions.

**Table 2:** IEEE CEC 2017 benchmark function specifications.

Function equation	Dim	Optimum
$\:{f}_{1}\left(x\right)={x}_{1}^{2}+1{0}^{6}{\sum\:}_{i=2}^{D}{x}_{i}^{2}$	30	100
$\:{f}_{2}\left(x\right)={\sum\:}_{i=1}^{D}\left|{x}_{i}^{2}\right|$	30	200
$\:{f}_{3}\left(x\right)={\sum\:}_{i=1}^{D}{x}_{i}^{2}+{\left({\sum\:}_{i=1}^{D}0.5{x}_{i}^{2}\right)}^{2}+{\left({\sum\:}_{i=1}^{D}0.5{x}_{i}^{2}\right)}^{4}$	30	300
$\:{f}_{4}\left(x\right)={\sum\:}_{i=1}^{D-1}\left(100{\left({x}_{i}^{2}-{x}_{i+1}\right)}^{2}+{\left({x}_{i}-1\right)}^{2}\right)$	30	400
$\:{f}_{5}\left(x\right)={\sum\:}_{i=1}^{D}\left({x}_{i}^{2}-10{cos}\left(2\pi\:{x}_{i}\right)+10\right)$	30	500
$\:{f}_{6}\left(x\right)=g\left({x}_{1},{x}_{2}\right)+g\left({x}_{2},{x}_{3}\right)+\dots\:+g\left({x}_{\text{D-}1},{x}_{D}\right)+g\left({x}_{D},{x}_{1}\right)$	30	600
$$\:g\left(x,y\right)=0.5+\frac{\left({{sin}}^{2}\left(\sqrt{{x}^{2}+{y}^{2}}\right)-0.5\right)}{{\left(1+0.001\left({x}^{2}+{y}^{2}\right)\right)}^{2}}$$		
$${f_7}\left( x \right) = \min \left( {\mathop \sum \nolimits_{i = 1}^D {{\left( {{{\overset{\lower0.5em\hbox{$\smash{\scriptscriptstyle\frown}$}}{x} }_i} - {\mu _0}} \right)}^2},dD + s\mathop \sum \nolimits_{i = 1}^D {{\left( {{{\overset{\lower0.5em\hbox{$\smash{\scriptscriptstyle\frown}$}}{x} }_i} - {\mu _1}} \right)}^2}} \right) + 10\left( {D - \mathop \sum \nolimits_{i = 1}^D \cos \left( {2\pi {{\overset{\lower0.5em\hbox{$\smash{\scriptscriptstyle\frown}$}}{z} }_i}} \right)} \right)$$	30	700
$\:{f}_{8}\left(x\right)={\sum\:}_{i=1}^{D}\left({z}_{i}^{2}-10{cos}\left(2\pi\:{z}_{i}\right)+10\right)+{{f}_{13}}^{*}$	30	800
$\:{f}_{9}\left(x\right)={{sin}}^{2}\left(\pi\:{w}_{1}\right)+{\sum\:}_{i=1}^{D}{\left({w}_{i}-1\right)}^{2}\left[1+10{{sin}}^{2}\left(\pi\:{w}_{i}+1\right)\right]+{\left({w}_{D}-1\right)}^{2}\left[1+{{sin}}^{2}\left(2\pi\:{w}_{D}\right)\right]$	30	900
$\:{f}_{10}\left(x\right)=418.9829\times\:D-{\sum\:}_{i=1}^{D}g\left({z}_{i}\right)\:,\:{z}_{i}={x}_{i}+4.209687462275036e+002$	30	1000
$\:{f}_{11}\left(x\right)={{\sum\:}_{i=1}^{D}\left(1{0}^{6}\right)}^{\frac{i-1}{D-1}}{x}_{i}^{2}$	3	1100
$\:{f}_{12}\left(x\right)=1{0}^{6}{x}_{1}^{2}+{\sum\:}_{i=2}^{D}{x}_{i}^{2}$	3	1200
$\:{f}_{13}\left(x\right)=-20{exp}\left(-0.2\sqrt{\frac{1}{D}{\sum\:}_{i=1}^{D}{x}_{i}^{2}}\right)-{exp}\left(\frac{1}{D}{\sum\:}_{i=1}^{D}{cos}\left(2\pi\:{x}_{i}\right)\right)+20+e$	3	1300
$\:{f}_{14}\left(x\right)={\sum\:}_{i=1}^{D}\left({\sum\:}_{k=0}^{kmax}\left[{a}^{k}{cos}\left(2\pi\:{b}^{k}(x+0.5\right))\right]\right)-D{\sum\:}_{k=0}^{kmax}\left[{a}^{k}{cos}\left(2\pi\:{b}^{k}.0.5\right)\right]$	4	1400
$\:{f}_{15}\left(x\right)={\sum\:}_{i=1}^{D}\frac{{x}_{i}^{2}}{4000}-{\prod\:}_{i=1}^{D}{cos}\left(\frac{{x}_{i}}{\sqrt{i}}\right)+1$	4	1500
$\:{f}_{16}\left(x\right)=\frac{10}{{D}^{2}}{{\prod\:}_{i=1}^{D}\left(1+i{\sum\:}_{j=1}^{32}\frac{\left|{2}^{j}{x}_{i}-round\left({2}^{j}{x}_{i}\right)\right|}{{2}^{j}}\right)}^{\frac{10}{{D}^{1.2}}}-\frac{10}{{D}^{2}}$	4	1600
$\:{f}_{17}\left(x\right)={\left|{\sum\:}_{i=1}^{D}{x}_{i}^{2}-D\right|}^{1/4}+\left(0.5{\sum\:}_{i=1}^{D}{x}_{i}^{2}+{\sum\:}_{i=1}^{D}{x}_{i}\right)/D+0.5$	5	1700
$\:{f}_{18}\left(x\right)={\left|{\left({\sum\:}_{i=1}^{D}{x}_{i}^{2}\right)}^{2}-{\left({\sum\:}_{i=1}^{D}{x}_{i}\right)}^{2}\right|}^{1/4}+\left(0.5{\sum\:}_{i=1}^{D}{x}_{i}^{2}+{\sum\:}_{i=1}^{D}{x}_{i}\right)/D+0.5$	5	1800
$\:{f}_{19}\left(x\right)={f}_{7}\left({f}_{4}\left({x}_{1},{x}_{2}\right)\right)+{f}_{7}\left({f}_{4}\left({x}_{2},{x}_{3}\right)\right)+\dots\:+{f}_{7}\left({f}_{4}\left({x}_{D-1},{x}_{D}\right)\right)+{f}_{7}\left({f}_{4}\left({x}_{D},{x}_{1}\right)\right)$	5	1900
$\:{f}_{20}\left(x\right)={\left[\frac{1}{D-1}{\sum\:}_{i=1}^{D-1}\left(\sqrt{{s}_{i}}\cdot\:\left({sin}\left(50.0{s}_{i}^{0.2}\right)+1\right)\right)\right]}^{2},{s}_{i}=\sqrt{{x}_{i}^{2}+{x}_{i+1}^{2}}$	6	2000
$\:{f}_{21}\left(x\right)={f}_{1}\left(M\left(x-{o}_{1}\right)\right)+{{f}_{21}}^{*}$	3	2100
$\:{f}_{22}\left(x\right)={f}_{2}\left(M\left(x-{o}_{2}\right)\right)+{{f}_{22}}^{*}$	3	2200
$\:{f}_{23}\left(x\right)={f}_{3}\left(M\left(x-{o}_{3}\right)\right)+{{f}_{23}}^{*}$	4	2300
$\:{f}_{24}\left(x\right)={f}_{4}\left(M\left(\frac{2.048\left(x-{o}_{4}\right)}{100}\right)+1\right)+{{f}_{24}}^{*}$	4	2400
$\:{f}_{25}\left(x\right)={f}_{5}\left(M\left(x-{o}_{5}\right)\right)+{{f}_{25}}^{*}$	5	2500
$\:{f}_{26}\left(x\right)={f}_{20}\left(M\left(\frac{2.048\left(x-{o}_{6}\right)}{100}\right)\right)+{{f}_{26}}^{*}$	5	2600
$\:{f}_{27}\left(x\right)={f}_{7}\left(M\left(\frac{600\left(x-{o}_{7}\right)}{100}\right)\right)+{{f}_{27}}^{*}$	6	2700
$\:{f}_{28}\left(x\right)={f}_{8}\left(\frac{5.12\left(x-{o}_{8}\right)}{100}\right)+{{f}_{28}}^{*}$	6	2800
$\:{f}_{29}\left(x\right)={f}_{9}\left(M\left(\frac{5.12\left(x-{o}_{9}\right)}{100}\right)\right)+{{f}_{29}}^{*}$	3	2900
$\:{f}_{30}\left(x\right)={f}_{30}\left(M\left(\frac{1000\left(x-{o}_{10}\right)}{100}\right)\right)+{{f}_{30}}^{*}$	3	3000

#### IEEE CEC 2022 benchmark functions

Table 3 shows the details of the IEEE CEC 2022 benchmark functions.

**Table 3:** IEEE CEC 2022 benchmark function specifications.

Functions	Describe	$\:{f}_{i}$
F1	Full rotated Zakharov	300
F2	Full rotated Rosenbrock	400
F3	Full rotated expanded Schaffer’s f6	600
F4	Shifted and full rotated non-continuous restrain	800
F5	Shifted and full rotated levy	900
F6	Hybrid	1800
F7	Hybrid	2000
F8	Hybrid	2200
F9	Composition	2300
F10	Composition	2400
F11	Composition	2600
F12	Composition	2700

### Ablation analysis

This section provides an in-depth analysis of the performance improvements introduced by the two enhancement strategies in RDFOA, validated through ablation experiments—a fundamental approach in scientific investigation. Such experiments are critical for assessing the robustness and reliability of research findings. By selectively removing specific components and evaluating the resulting changes in performance, researchers can isolate the individual effects of each mechanism, thereby excluding alternative explanations. This process clarifies the distinct contribution of each component, ensuring that the conclusions drawn are both valid and reproducible.

Ablation testing is essential for hypothesis validation, enhancing the credibility of the research while minimizing the impact of confounding factors. The results of these experiments are summarized in Tables 4 and 5. Here, RFOA denotes the FOA variant modified solely by the random spare strategy, whereas DFOA applies only the double adaptive weighting mechanism. A total of 30 independent runs were conducted on benchmark functions from the CEC 2017 test suite. The data indicate that RDFOA, which integrates both enhancement mechanisms, consistently outperforms the singly improved variants. Specifically, RDFOA achieved superior results on eight functions compared to RFOA and on twenty functions compared to DFOA, highlighting the effectiveness of the combined strategy in advancing FOA—an improvement unattainable by either enhancement alone.

**Table 4:** Detailed data of ablation analysis.

	RDFOA		RFOA		DFOA		FOA	
	Average	Std	Average	Std	Average	Std	Average	Std
Func 1	3.3830E + 03	3.5416E + 03	2.2984E + 03	2.5187E + 03	8.7287E + 03	1.5405E + 04	8.7895E + 05	4.9640E + 05
Func 2	6.9920E + 06	3.8146E + 07	1.0429E + 03	4.1550E + 03	1.9898E + 14	1.0712E + 15	5.1811E + 15	2.5203E + 16
Func 3	4.9419E + 03	2.1655E + 03	3.0409E + 02	1.4282E + 01	1.1823E + 04	3.9116E + 03	1.7774E + 03	2.5689E + 03
Func 4	4.7725E + 02	2.1689E + 01	4.6737E + 02	3.1303E + 01	5.0235E + 02	1.9782E + 01	5.0897E + 02	2.7516E + 01
Func 5	5.4324E + 02	1.0200E + 01	5.4607E + 02	1.1934E + 01	5.9711E + 02	4.3871E + 01	5.3621E + 02	1.0452E + 01
Func 6	6.0002E + 02	4.3473E-02	6.0007E + 02	1.9358E-01	6.0000E + 02	3.3056E-03	6.0009E + 02	2.5348E-01
Func 7	7.8476E + 02	3.3982E + 01	7.8347E + 02	2.2203E + 01	8.5745E + 02	1.8734E + 01	7.7122E + 02	1.0730E + 01
Func 8	8.4721E + 02	1.7157E + 01	8.4788E + 02	1.3448E + 01	8.7806E + 02	4.3725E + 01	8.3647E + 02	1.0369E + 01
Func 9	9.0293E + 02	3.0480E + 00	9.1583E + 02	2.1563E + 01	9.0087E + 02	7.5810E-01	9.2645E + 02	3.4474E + 01
Func 10	4.7660E + 03	1.5214E + 03	3.5185E + 03	4.8844E + 02	5.6867E + 03	1.1948E + 03	3.1433E + 03	7.3549E + 02
Func 11	1.1309E + 03	2.4538E + 01	1.1433E + 03	3.0920E + 01	1.1607E + 03	3.2342E + 01	1.8331E + 03	1.2484E + 03
Func 12	2.3960E + 05	2.2181E + 05	2.8849E + 05	2.5920E + 05	1.2039E + 06	7.3988E + 05	2.5820E + 06	2.1011E + 06
Func 13	1.8311E + 04	1.6589E + 04	1.2564E + 04	1.3245E + 04	2.7066E + 04	3.6853E + 04	1.2031E + 06	6.4125E + 05
Func 14	5.9257E + 04	6.7866E + 04	9.2302E + 04	8.3992E + 04	4.0925E + 05	5.4749E + 05	1.2253E + 06	1.7345E + 06
Func 15	6.9461E + 03	8.5988E + 03	9.1191E + 03	9.1516E + 03	2.2274E + 04	8.0498E + 04	3.6282E + 04	5.8068E + 04
Func 16	2.3774E + 03	2.7893E + 02	2.4229E + 03	3.5202E + 02	2.1926E + 03	2.4230E + 02	2.2620E + 03	3.2154E + 02
Func 17	2.0290E + 03	2.1273E + 02	2.1106E + 03	2.3100E + 02	1.9383E + 03	1.1625E + 02	2.1610E + 03	2.1366E + 02
Func 18	2.3369E + 05	1.6732E + 05	3.4256E + 05	2.8974E + 05	4.2091E + 05	3.5901E + 05	2.1674E + 06	3.1293E + 06
Func 19	1.1350E + 04	1.2826E + 04	1.2476E + 04	1.3204E + 04	8.9198E + 03	9.6549E + 03	8.4842E + 04	9.9567E + 04
Func 20	2.3053E + 03	1.4960E + 02	2.4314E + 03	1.6788E + 02	2.3053E + 03	1.6617E + 02	2.3588E + 03	1.7647E + 02
Func 21	2.3470E + 03	2.1680E + 01	2.3478E + 03	1.5022E + 01	2.3945E + 03	4.2478E + 01	2.3372E + 03	1.0875E + 01
Func 22	2.3004E + 03	9.6987E-01	3.0337E + 03	1.2963E + 03	2.3013E + 03	2.2394E + 00	2.8227E + 03	9.5942E + 02
Func 23	2.6976E + 03	5.4038E + 01	2.7196E + 03	1.6411E + 01	2.6961E + 03	1.6432E + 01	2.7070E + 03	1.6650E + 01
Func 24	2.8739E + 03	1.3875E + 01	2.8863E + 03	1.8843E + 01	2.9552E + 03	4.2677E + 01	2.8750E + 03	1.3975E + 01
Func 25	2.8925E + 03	1.2471E + 01	2.8933E + 03	1.3094E + 01	2.8893E + 03	4.3242E + 00	2.8930E + 03	1.2402E + 01
Func 26	4.0261E + 03	3.4898E + 02	4.1317E + 03	3.9046E + 02	3.9353E + 03	2.5464E + 02	3.9959E + 03	4.8351E + 02
Func 27	3.2257E + 03	1.5146E + 01	3.2400E + 03	1.6245E + 01	3.2327E + 03	1.3566E + 01	3.2439E + 03	1.7091E + 01
Func 28	3.1764E + 03	5.9346E + 01	3.1724E + 03	6.2302E + 01	3.2211E + 03	1.5315E + 01	3.2292E + 03	2.1396E + 01
Func 29	3.6289E + 03	1.4080E + 02	3.7149E + 03	1.7148E + 02	3.4938E + 03	1.6370E + 02	3.7889E + 03	2.3337E + 02
Func 30	1.0360E + 04	3.6331E + 03	1.0952E + 04	3.4288E + 03	1.2622E + 04	5.3237E + 03	1.9705E + 05	2.0758E + 05

**Table 5:** Ablation analysis.

Algorithm	Rank	+/=/-	AVG
RDFOA	1	~	1.7
RFOA	2	9/2/19	2.7
DFOA	3	20/5/5	2.6
FOA	4	14/3/13	2.8

### Scalability analysis

This section evaluates the scalability of RDFOA by applying it to optimization problems of varying dimensionalities. Scalability analysis is a crucial approach for assessing how effectively an evolutionary algorithm adapts to increasing problem size and complexity. By systematically varying the number of dimensions, we evaluate the algorithm’s ability to maintain efficiency and performance under diverse computational demands. These tests consider factors such as computational cost, execution time, and solution accuracy, providing insights into the practical feasibility and limitations of RDFOA when addressing large-scale optimization tasks.

Such evaluations are essential for validating the algorithm’s applicability in real-world scenarios, as scalable algorithms are better equipped to handle complex, high-dimensional problems commonly encountered in industrial and engineering fields. In this study, three widely used benchmark dimensions—30, 50, and 100—are selected to assess RDFOA’s global optimization performance across different scales. The results, summarized in Table 6, demonstrate that RDFOA consistently outperforms the original Arithmetic Optimization Algorithm (AO) across all tested dimensions. The inclusion of AO in the comparison further underscores RDFOA’s robustness and adaptability in managing increasingly complex optimization problems.

**Table 6:** Scalability tests in three dimensions.

	Dim	30		50		100	
	Metric	RDFOA	FOA	RDFOA	FOA	RDFOA	FOA
F1	Avg	4.30000E + 03	5.10000E + 03	6.00000E + 03	6.90000E + 03	7.20000E + 03	1.10000E + 04
	STD	1.00000E + 06	5.80000E + 05	2.40000E + 06	7.80000E + 05	5.10000E + 06	1.30000E + 06
F2	Avg	1.00000E + 07	5.00000E + 07	7.20000E + 27	3.90000E + 28	7.20000E + 77	3.90000E + 78
	STD	6.90000E + 15	3.70000E + 16	8.50000E + 30	4.60000E + 31	1.20000E + 98	6.30000E + 98
F3	Avg	4.90000E + 03	2.60000E + 03	4.90000E + 04	1.20000E + 04	2.40000E + 05	3.30000E + 04
	STD	1.90000E + 03	3.40000E + 03	1.00000E + 04	7.90000E + 03	8.00000E + 04	2.10000E + 04
F4	Avg	4.80000E + 02	2.40000E + 01	5.00000E + 02	5.40000E + 01	6.40000E + 02	2.90000E + 01
	STD	4.90000E + 02	3.10000E + 01	5.60000E + 02	4.90000E + 01	6.50000E + 02	3.40000E + 01
F5	Avg	5.50000E + 02	2.70000E + 01	6.40000E + 02	9.20000E + 01	1.20000E + 03	1.40000E + 02
	STD	5.40000E + 02	1.10000E + 01	5.80000E + 02	3.50000E + 01	1.10000E + 03	6.30000E + 01
F6	Avg	6.00000E + 02	1.50000E-02	6.00000E + 02	9.20000E-03	6.00000E + 02	1.20000E-02
	STD	6.00000E + 02	3.20000E-02	6.00000E + 02	2.10000E-01	6.00000E + 02	6.80000E-02
F7	Avg	7.90000E + 02	3.30000E + 01	1.00000E + 03	7.60000E + 01	1.60000E + 03	1.30000E + 02
	STD	7.70000E + 02	2.50000E + 01	9.30000E + 02	9.40000E + 01	1.50000E + 03	7.70000E + 01
F8	Avg	8.40000E + 02	1.00000E + 01	9.30000E + 02	8.00000E + 01	1.50000E + 03	1.60000E + 02
	STD	8.40000E + 02	9.30000E + 00	9.10000E + 02	7.30000E + 01	1.40000E + 03	1.30000E + 02
F9	Avg	9.00000E + 02	5.10000E + 00	9.10000E + 02	5.20000E + 00	9.10000E + 02	5.40000E + 00
	STD	9.40000E + 02	1.40000E + 02	9.80000E + 02	8.30000E + 01	1.10000E + 03	9.20000E + 01
F10	Avg	4.50000E + 03	1.50000E + 03	9.20000E + 03	2.70000E + 03	2.60000E + 04	5.70000E + 03
	STD	3.20000E + 03	5.40000E + 02	6.30000E + 03	2.50000E + 03	2.40000E + 04	2.00000E + 03
F11	Avg	1.10000E + 03	2.60000E + 01	1.20000E + 03	2.70000E + 01	1.60000E + 03	1.80000E + 02
	STD	1.40000E + 03	2.70000E + 02	1.20000E + 03	9.70000E + 01	2.40000E + 03	9.90000E + 02
F12	Avg	2.10000E + 05	2.20000E + 05	1.80000E + 06	1.00000E + 06	2.70000E + 06	1.10000E + 06
	STD	3.00000E + 06	2.10000E + 06	1.00000E + 07	3.70000E + 06	2.80000E + 07	1.10000E + 07
F13	Avg	1.80000E + 04	2.00000E + 04	6.90000E + 03	7.40000E + 03	5.40000E + 03	4.60000E + 03
	STD	1.30000E + 06	1.50000E + 06	2.40000E + 06	1.10000E + 06	4.00000E + 05	2.70000E + 05
F14	Avg	4.50000E + 04	3.80000E + 04	8.60000E + 04	6.30000E + 04	2.70000E + 05	9.60000E + 04
	STD	7.30000E + 05	1.10000E + 06	2.30000E + 06	1.80000E + 06	3.40000E + 06	1.70000E + 06
F15	Avg	5.90000E + 03	5.10000E + 03	9.90000E + 03	6.50000E + 03	4.10000E + 03	3.60000E + 03
	STD	4.50000E + 04	7.90000E + 04	2.20000E + 05	2.70000E + 05	2.80000E + 05	3.10000E + 05
F16	Avg	2.40000E + 03	2.70000E + 02	2.90000E + 03	4.10000E + 02	6.10000E + 03	2.00000E + 03
	STD	2.40000E + 03	2.50000E + 02	2.70000E + 03	3.80000E + 02	4.90000E + 03	1.80000E + 03
F17	Avg	2.00000E + 03	2.10000E + 02	2.60000E + 03	3.10000E + 02	5.30000E + 03	9.20000E + 02
	STD	2.10000E + 03	1.90000E + 02	2.40000E + 03	2.90000E + 02	4.40000E + 03	1.10000E + 03
F18	Avg	2.40000E + 05	2.30000E + 05	1.30000E + 06	1.20000E + 06	2.80000E + 06	1.50000E + 06
	STD	1.30000E + 06	1.60000E + 06	3.90000E + 06	6.10000E + 06	3.20000E + 06	2.00000E + 06
F19	Avg	1.40000E + 04	1.50000E + 04	1.60000E + 04	1.10000E + 04	5.10000E + 03	2.80000E + 03
	STD	7.60000E + 04	1.40000E + 05	8.50000E + 04	1.10000E + 05	3.10000E + 05	2.20000E + 05
F20	Avg	2.30000E + 03	1.60000E + 02	2.70000E + 03	2.80000E + 02	5.60000E + 03	1.00000E + 03
	STD	2.40000E + 03	2.20000E + 02	2.50000E + 03	2.40000E + 02	4.50000E + 03	1.00000E + 03
F21	Avg	2.30000E + 03	1.60000E + 01	2.50000E + 03	8.70000E + 01	3.00000E + 03	1.20000E + 02
	STD	2.30000E + 03	8.50000E + 00	2.40000E + 03	6.90000E + 01	3.00000E + 03	4.40000E + 01
F22	Avg	2.30000E + 03	1.20000E + 00	1.00000E + 04	3.30000E + 03	2.80000E + 04	9.30000E + 03
	STD	2.80000E + 03	9.30000E + 02	8.60000E + 03	3.30000E + 03	2.40000E + 04	6.20000E + 03
F23	Avg	2.70000E + 03	1.40000E + 01	2.80000E + 03	2.10000E + 01	3.00000E + 03	3.20000E + 01
	STD	2.70000E + 03	1.20000E + 01	2.80000E + 03	3.00000E + 01	3.10000E + 03	4.70000E + 01
F24	Avg	2.90000E + 03	2.50000E + 01	3.10000E + 03	1.00000E + 02	3.60000E + 03	6.70000E + 01
	STD	2.90000E + 03	1.70000E + 01	3.10000E + 03	8.00000E + 01	3.60000E + 03	5.70000E + 01
F25	Avg	2.90000E + 03	6.90000E + 00	3.00000E + 03	3.60000E + 01	3.20000E + 03	6.00000E + 01
	STD	2.90000E + 03	1.90000E + 01	3.10000E + 03	3.20000E + 01	3.30000E + 03	5.00000E + 01
F26	Avg	3.90000E + 03	5.00000E + 02	4.80000E + 03	4.60000E + 02	9.70000E + 03	2.10000E + 03
	STD	4.10000E + 03	3.70000E + 02	4.60000E + 03	6.40000E + 02	8.40000E + 03	6.70000E + 02
F27	Avg	3.20000E + 03	1.60000E + 01	3.40000E + 03	7.00000E + 01	3.50000E + 03	6.10000E + 01
	STD	3.20000E + 03	1.50000E + 01	3.50000E + 03	1.00000E + 02	3.50000E + 03	6.80000E + 01
F28	Avg	3.20000E + 03	5.10000E + 01	3.30000E + 03	2.10000E + 01	3.40000E + 03	2.40000E + 01
	STD	3.20000E + 03	2.40000E + 01	3.30000E + 03	2.30000E + 01	3.40000E + 03	3.50000E + 01
F29	Avg	3.70000E + 03	1.90000E + 02	3.80000E + 03	3.50000E + 02	5.50000E + 03	5.40000E + 02
	STD	3.70000E + 03	2.30000E + 02	3.80000E + 03	2.60000E + 02	5.00000E + 03	3.30000E + 02
F30	Avg	9.20000E + 03	4.90000E + 03	1.10000E + 06	3.40000E + 05	1.30000E + 04	6.40000E + 03
	STD	1.60000E + 05	1.30000E + 05	1.20000E + 06	3.40000E + 05	1.20000E + 06	6.60000E + 05
+/-/=	~	~	18/6/6	~	15/7/8	~	16/9/5

**Fig. 2:**
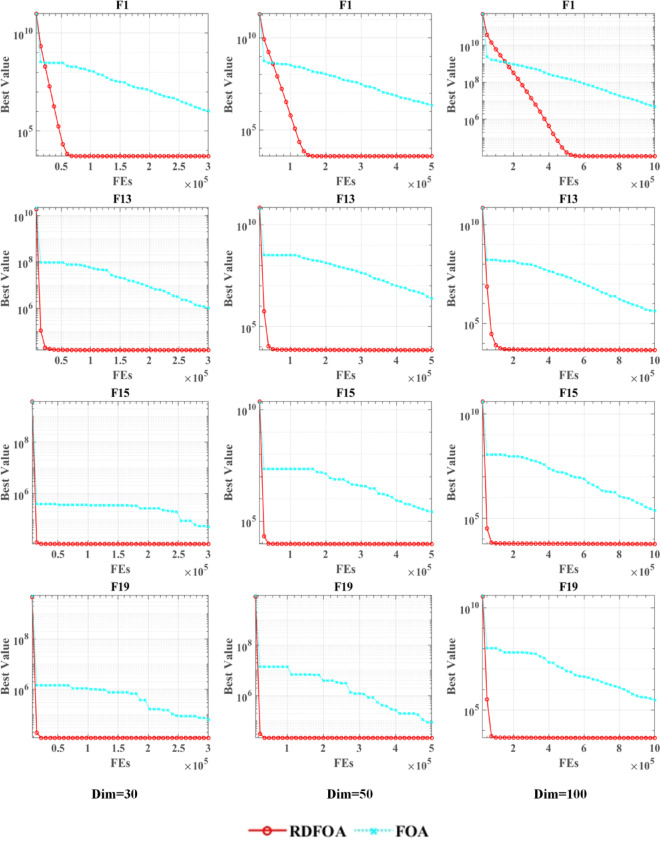
Scalability analysis on the IEEE CEC 2017 benchmark functions.

Figure 2 displays the convergence patterns of RDFOA and AO for various test functions, with red curves for RDFOA and blue curves for AO. The dimensions displayed are 30, 50, and 100, and the test functions are F1, F13, F15, and F19 from the CEC 2017 benchmark set. It is evident from the figure that RDFOA outperforms AO in terms of convergence speed and accuracy.

### Historical searches

Visual analysis of algorithmic search behavior plays a pivotal role in evolutionary computation research. Through graphical representations, researchers gain intuitive insights into search dynamics, including trajectory patterns, convergence speed, and the ability to escape local optima within the solution space. Such visual tools enhance understanding of algorithmic mechanisms and provide critical information for identifying performance bottlenecks and opportunities for improvement. Moreover, visual diagnostics are instrumental in revealing potential weaknesses, guiding targeted refinements, and ultimately facilitating more effective algorithm design. Therefore, incorporating visual experimentation into performance analysis is essential for both theoretical investigation and practical advancement of evolutionary algorithms.

**Fig. 3:**
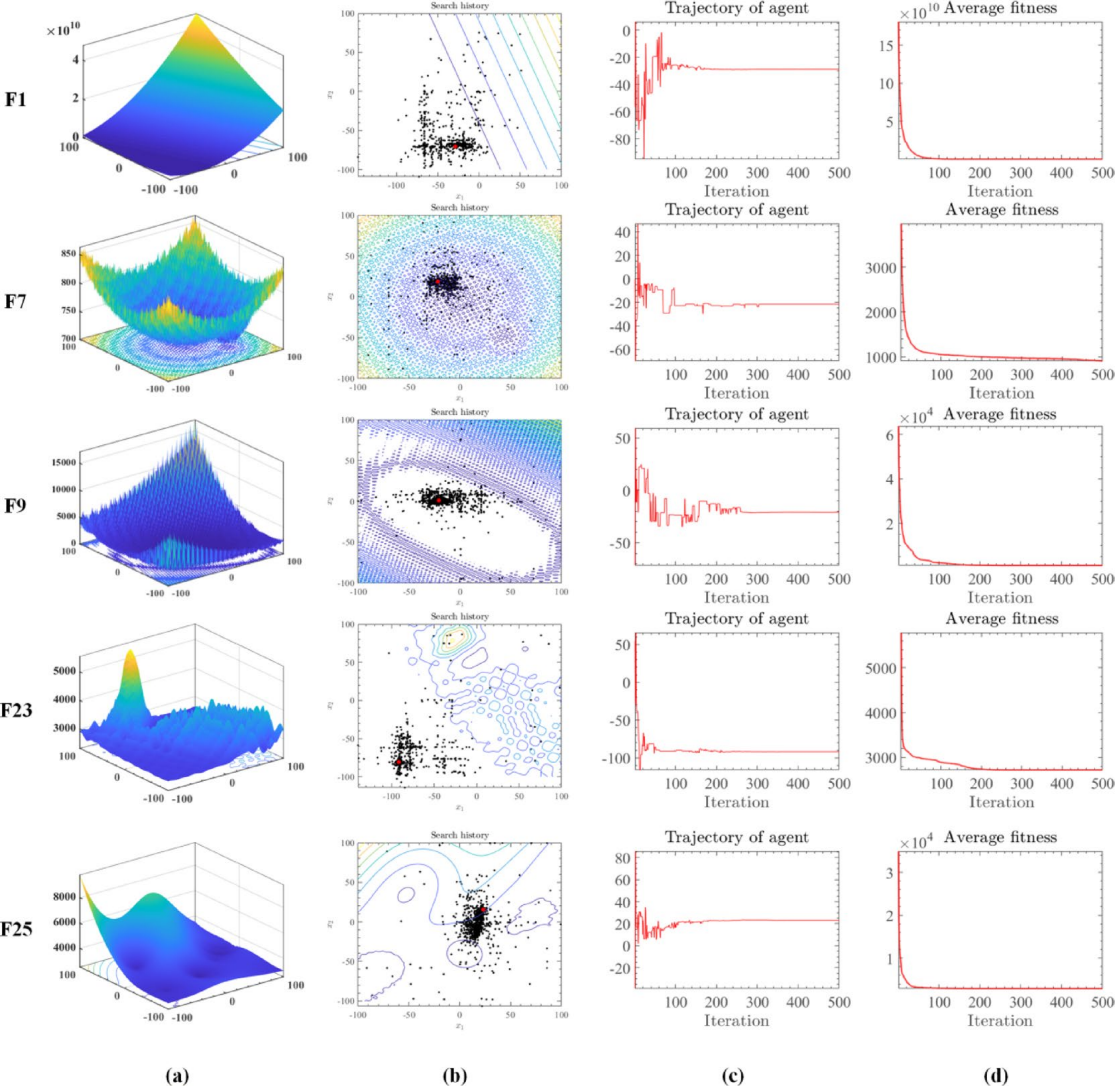
Evolutionary trajectory of RDFOA on the IEEE CEC 2017 benchmark functions.

To illustrate the search behavior of RDFOA, Fig. 3 presents detailed visualizations based on benchmark functions from the IEEE CEC 2017 suite, specifically functions F1, F7, F9, F23, and F25. Figure 3a depicts simulated landscapes of these functions, providing contextual insight into their complexity. Figure 3b traces the historical search trajectory of RDFOA, where red points indicate global optima and black points represent positions identified by RDFOA during successive iterations. The convergence pattern demonstrates that RDFOA consistently approaches the global optimum while effectively avoiding local traps. The dispersion of black points—both near the red optima and throughout the search space—reflects the algorithm’s strong global exploration capability.

Figure 3c illustrates the relative error with respect to the global optimum over iterations, showing that RDFOA generally stabilizes around the 500th iteration. Meanwhile, Fig. 3d depicts the evolution of average fitness values across generations, exhibiting an overall downward trend indicative of continuous optimization progress.

### Comparison of other related algorithms

#### Comparative experiments at CEC 2017 benchmark functions

This section evaluates RDFOA using the IEEE CEC 2017 benchmark functions. The Wilcoxon signed-rank test^27^ and Friedman test^28^ was employed to evaluate performance. To ensure fair comparisons, all algorithms were evaluated under the same conditions. The population size (popsize) was set to 30, the number of sub-populations was fixed at 3. The maximum number of evaluations (MaxFEs) is uniformly set to 300,000. In order to reduce the impact of randomness, each algorithm is independently run 30 times on each benchmark function. In addition, to compare the performance of various algorithms in benchmark tests, the Friedman test method was used to rank the algorithms based on their average performance. This testing method not only provides a statistically significant comparison, but also outputs the average ranking of each algorithm. In the table, the optimal results of each test function are highlighted in bold for readers to more intuitively identify the best performance.

Table 7 presents a detailed comparison of RDFOA against various alternative algorithms. RDFOA achieves the highest ranking with an impressively low average score of 1.23E + 00, demonstrating its outstanding optimization capability. The “~” symbol in the +/=/- column indicates that RDFOA serves as the reference algorithm and is not directly compared against itself. Its consistently lower average score across benchmark functions underscores RDFOA’s robustness and efficiency in optimization tasks.

In comparison, HGWO ranks ninth with a significantly higher average score of 9.11E + 00. The +/=/- metric of 30/0/0 indicates that RDFOA outperforms HGWO in all instances, highlighting the relative inefficiency of HGWO in these tasks. Similar trends are observed for SCADE and CCMWOA, ranked 11th and 12th respectively, each with an average score exceeding 1.17E + 01 and no wins against RDFOA. These results emphasize their inferior performance compared to RDFOA.

Algorithms such as QCSCA and CLACO, ranked third and second respectively, demonstrate stronger optimization capabilities, with average scores of 3.65E + 00 (+/=/-: 19/4/7) and 2.75E + 00 (+/=/-: 17/8/5). Although these algorithms occasionally outperform RDFOA, they consistently fall short of matching RDFOA’s overall dominance. Similarly, BLPSO and GCHHO, ranked fourth and fifth, with average scores of 3.89E + 00 (+/=/-: 21/1/8) and 5.54E + 00 (+/=/-: 20/2/8), exhibit intermittent competitive performance but remain inferior to RDFOA overall.

Finally, BWOA and CCMSCSA, ranked tenth and eighth respectively, show relatively poor performance, with average scores of 9.28E + 00 and 8.98E + 00. Their +/=/- metrics further confirm their inability to compete effectively with RDFOA.

In summary, the experimental results clearly demonstrate the significant advantages of RDFOA over other algorithms on the CEC 2017 benchmark suite. Its leading rank and lowest average error highlight the algorithm’s efficiency and stability in handling complex optimization problems. These findings further validate RDFOA’s potential as a versatile and powerful optimization tool across diverse application scenarios.

**Table 7:** Experiments comparing RDFOA with alternative competing algorithms on the IEEE CEC 2017 benchmark functions.

Algorithm	Rank	+/=/−	AVG
RDFOA	1	~	1.23E + 00
HGWO	9	30/0/0	9.11E + 00
WEMFO	6	25/0/5	5.75E + 00
mSCA	7	25/0/5	6.43E + 00
SCADE	11	30/0/0	1.17E + 01
CCMWOA	12	30/0/0	1.24E + 01
QCSCA	3	19/4/7	3.65E + 00
BWOA	10	30/0/0	9.28E + 00
CCMSCSA	8	29/0/1	8.98E + 00
CLACO	2	17/8/5	2.75E + 00
BLPSO	4	21/1/8	3.89E + 00
GCHHO	5	20/2/8	5.54E + 00

**Fig. 4:**
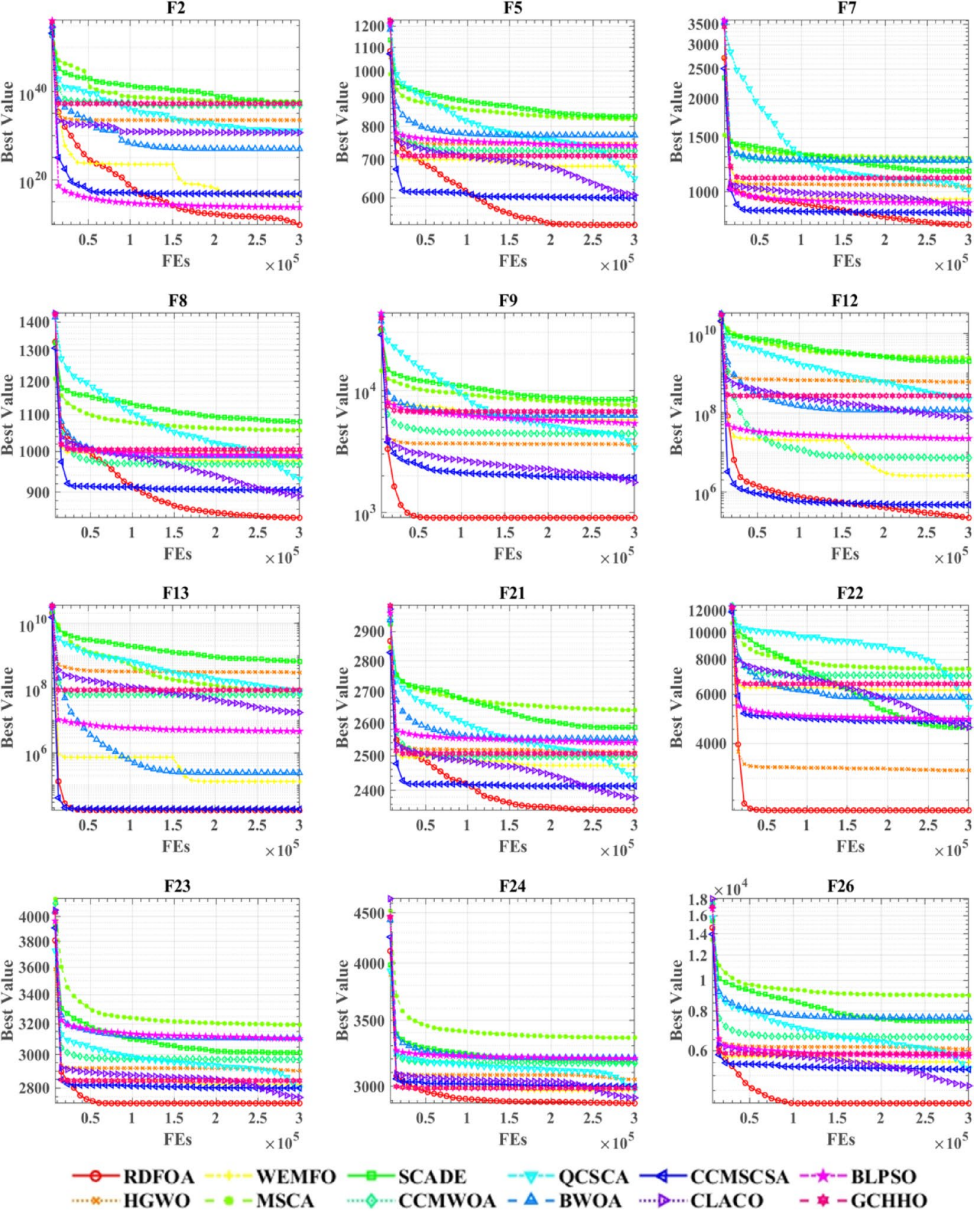
Performance comparisons of RDFOA with state-of-the-art competitors on the IEEE CEC 2017.

Figure 4 illustrates the convergence behavior of RDFOA in comparison with other algorithms on the CEC 2017 benchmark functions. In evolutionary algorithm research, convergence curves are essential for tracking the optimization trajectory over time. These graphical representations enable the evaluation of an algorithm’s convergence rate, stability, and ability to avoid common issues such as premature convergence or oscillatory behavior. By visualizing the progression of objective values across iterations, researchers can assess the effectiveness and efficiency of different methods and make informed adjustments to algorithmic parameters to enhance performance.

Convergence plots also serve as diagnostic tools to evaluate how well an algorithm adapts to varying problem complexities, making them indispensable for both algorithm design and performance assessment. In this study, convergence curves are presented for twelve test functions, with the x-axis representing the number of iterations and the y-axis indicating the corresponding objective values.

The results demonstrate that RDFOA exhibits distinct advantages in functions F5, F8, F22, and F26, achieving rapid convergence and maintaining the lowest objective values among the compared algorithms. Even on more challenging functions—where the performance curves of multiple algorithms tend to overlap—RDFOA consistently delivers competitive or superior optimization results, underscoring its robustness across diverse problem landscapes.

#### Comparative experiments at CEC 2022 benchmark functions

The competing algorithms involved in this experiment include HGWO^29^, WEMFO^30^, mSCA^31^, SCADE^32^, CCMWOA^33^, QCSCA^34^, BWOA^35^, CCMSCSA^36^, CLACO^37^, BLPSO^38^, GCHHO^39^.

Table 8 presents a comprehensive comparison of RDDOA with various other optimization methods based on IEEE CEC 2022 test functions. This table summarizes the average performance score (AVG) of each algorithm, its performance relationship with RDDOA (represented by+,=, -), and the final ranking results. Among them, “+” indicates that RDFOA performs better than the comparison algorithm, “-” indicates that it is slightly inferior, and “=” indicates that there is no significant statistical difference between the two. To verify the significance of the differences, this study used Wilcoxon signed rank test^27^ and Friedman rank test^28^.

The results demonstrate that RDFOA achieved the lowest mean score of 1.56E + 00, establishing itself as the best-performing optimizer in this experiment. The statistics in the “+/=/-” column further reflect its consistent superiority across multiple functions, confirming the robustness and efficiency of RDFOA in tackling complex optimization tasks.

Among the other algorithms, QCSCA ranks second with an average score of 2.87E + 00, closely following RDFOA. Although its overall score is slightly higher, the +/=/- indicator of 6/0/6 signifies advantages in certain tests, highlighting its strong competitiveness.

Additionally, HGWO, BWOA, and WEMFO rank ninth, eighth, and sixth respectively, with average scores of 7.34E + 00, 7.21E + 00, and 5.11E + 00. While these algorithms perform well on several benchmark functions (+/=/- values of 10/1, 9/2/1, and 9/2/1, respectively), their overall performance remains inferior to RDFOA, underscoring RDFOA’s superior stability and adaptability across diverse test scenarios.

Conversely, mSCA, SCADE, CCMWOA, and CCMCSSA—algorithms incorporating chaos strategies and diverse search mechanisms—rank seventh, twelfth, eleventh, and tenth, respectively. Their performance indicators reveal varying degrees of competitiveness relative to RDFOA, with alternating advantages and disadvantages observed across specific optimization tasks.

Overall, the experimental results convincingly demonstrate that RDFOA outperforms most comparative methods on the IEEE CEC 2022 benchmark suite, showcasing its high reliability and excellent adaptability in solving global optimization problems. These findings further validate RDFOA’s broad applicability in various engineering and scientific domains, positioning it as a core optimization tool in the field.

**Table 8:** Experiments comparing RDFOA with alternative competing algorithms at IEEE CEC 2022 benchmark functions.

Algorithm	Rank	+/=/−	AVG	
RDFOA	1	~	1.56E + 00	
HGWO	9	10/1/1	7.34E + 00	
mSCA	7	10/0/2	5.87E + 00	
WEMFO	6	9/2/1	5.11E + 00	
SCADE	12	10/2/0	8.45E + 00	
BWOA	8	9/2/1	7.21E + 00	
CCMSCSA	10	10/1/1	8.32E + 00	
CCMWOA	11	9/3/0	8.23E + 00	
QCSCA	2	6/0/6	2.87E + 00	
CLACO	3	4/2/6	3.44E + 00	
BLPSO	5	8/2/2	4.77E + 00	
GCHHO	4	7/2/3	4.44E + 00	

**Fig. 5:**
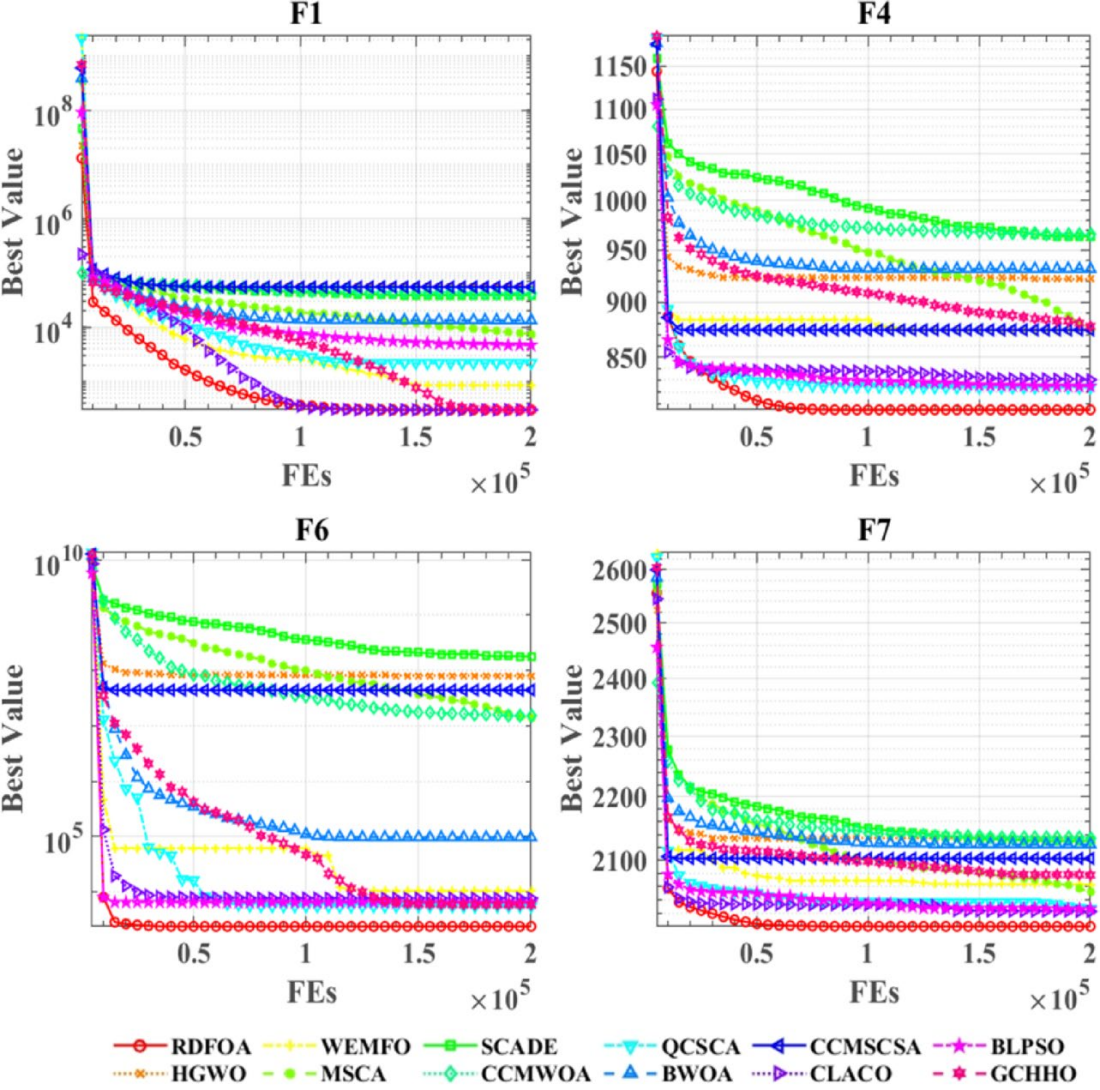
Performance comparisons of RDFOA with state-of-the-art competitors on the IEEE CEC 2022 benchmark functions.

Figure 5 illustrates the convergence performance of RDFOA in comparison with several competing algorithms on the CEC 2022 benchmark suite. The plot presents the convergence trends across nine test functions, with iterations represented along the x-axis and the corresponding objective values on the y-axis. RDFOA shows significant convergence superiority on functions F1, F4, F6, and F7, where it quickly reaches the optimal solutions and sustains the lowest objective values throughout the iterations. Furthermore, even in other more challenging cases—where the convergence curves of different algorithms are closely clustered—RDFOA consistently attains the best optimization results, demonstrating strong robustness and effectiveness.

## Application to oil reservoir production

Reservoir production optimization aims to determine the optimal operational settings for each well, with the primary goal of maximizing the net present value (NPV). However, as the number of wells and production periods increase, the complexity of the problem escalates dramatically due to the combinatorial growth in possible solution configurations, which leads to a substantial expansion in the dimensionality of the optimization variables. This classifies the problem as a typical NP-hard challenge, making it well-suited for the application of evolutionary algorithms.

In this section, we apply RDFOA to a three-channel reservoir model simulated using the Eclipse reservoir simulation software. The effectiveness of RDFOA is assessed by benchmarking its performance against several widely recognized evolutionary optimization algorithms. For the sake of experimental simplicity, nonlinear constraints commonly encountered in oilfield production are disregarded, focusing exclusively on maximizing the objective function—the NPV—as defined in Eq. 23.

23 $$\:NPV\left(x,z\right)=\sum\:_{t=1}^{n}\:{\Delta\:}t\frac{{Q}_{o,t}\cdot\:{r}_{o}-{Q}_{w,t}\cdot\:{r}_{w}-{Q}_{i,t}\cdot\:{r}_{i}}{{\left(1+b\right)}^{{p}_{t}}}$$

Here, $\:x$ represents the set of optimization variables, which in this experiment includes the injection and recovery rates for each well. The total simulation time is indicated by $\:n$, and $\:{Q}_{o,t}$, $\:{Q}_{w,t}$, $\:{Q}_{i,t}$ refer to the oil production rate, water production rate, and water injection rate, respectively, at the time step *t*. The term $\:{r}_{o}$ represents oil revenue, while $\:{r}_{w}$ and $\:{r}_{i}$ denote the costs associated with water treatment and injection. The parameter bbb indicates the average annual interest rate, and $\:{p}_{t}$ represents the number of years that have passed.

**Fig. 6:**
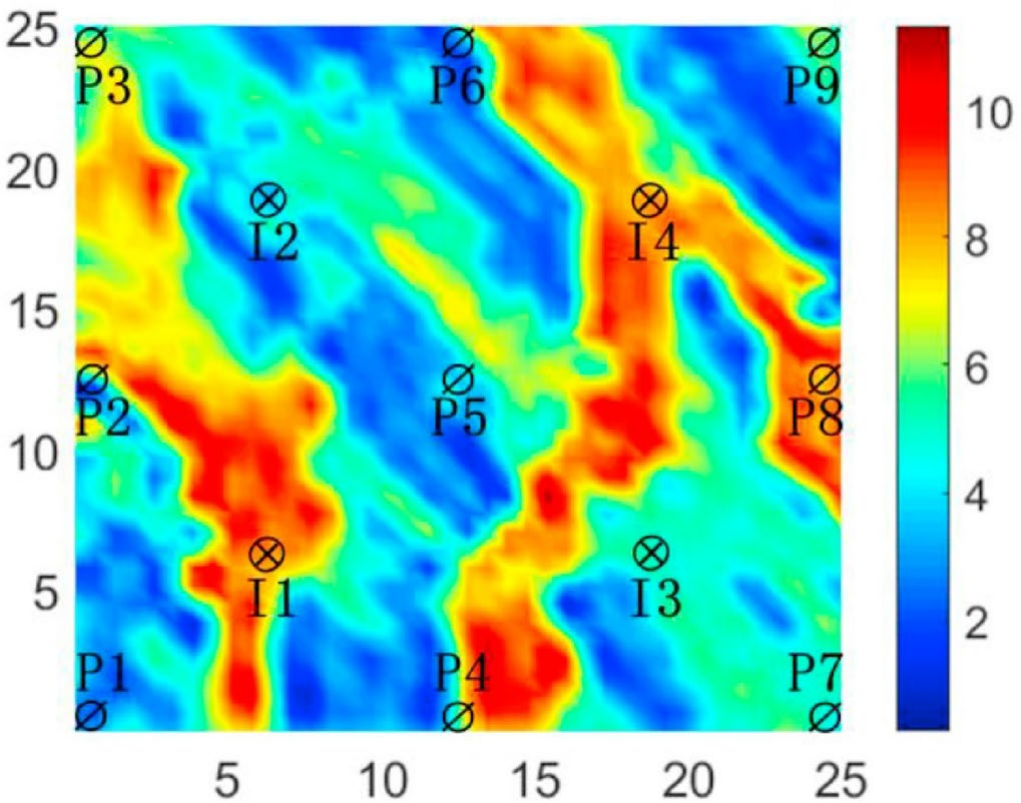
Log-permeability distribution of three-channel model.

This three-channel reservoir model is defined as a heterogeneous, two-dimensional reservoir with nine production wells and four injection wells placed in a 5-point arrangement. To ensure fair comparisons, all algorithms were evaluated under the same conditions. The population size (popsize) was set to 30, the number of sub-populations was fixed at 3. The maximum number of evaluations (MaxFEs) is uniformly set to 300,000. In order to reduce the impact of randomness, each algorithm is independently run 30 times on each benchmark function. The model employs a grid with dimensions of 25 * 25 * 1, where each grid block has a length of 100 feet. Each block is 20 feet thick, and the porosity is uniformly fixed at 0.2. The distribution of permeability within the model is shown in Fig. 6.

The production optimization task focuses on tuning variables such as the injection rates for injection wells and the fluid recovery rates for production wells. Specifically, water injection rates vary between 0 and 500 STB/DAY, while the extraction rates for production wells range from 0 to 200 STB/DAY. The thermal storage system is configured for a total operation period of 1800 days, divided into decision intervals of 360 days each, resulting in 65 decision variables. The objective function employed for this optimization is the net present value (NPV).

**Fig. 7:**
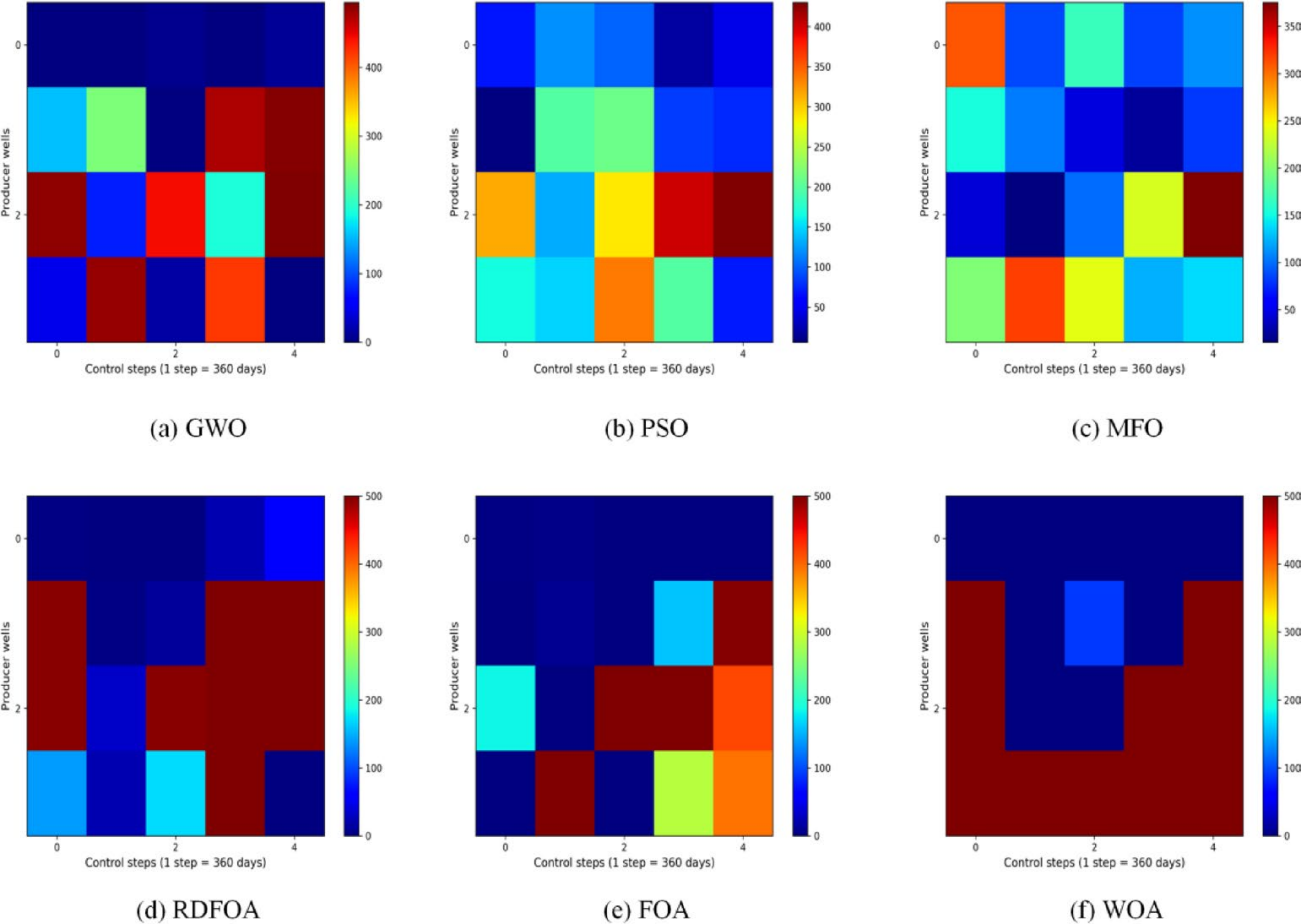
The optimal water-injection rate obtained by each algorithm for the three-channel model.

**Fig. 8:**
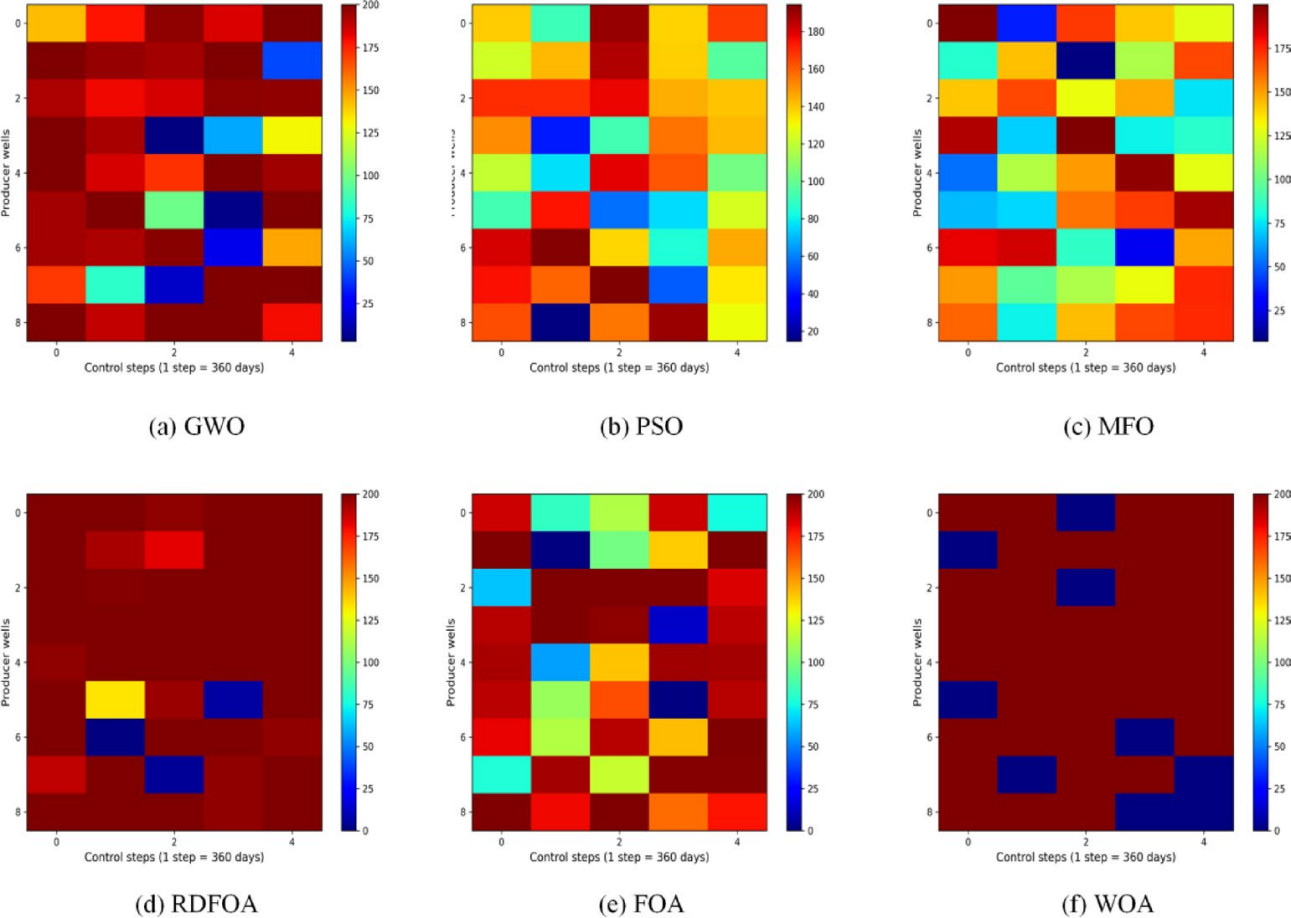
The optimal liquid-production rate obtained by each algorithm for the three-channel model.

Figures 7 and 8 depict the final optimized schedules for water injection and liquid production rates generated by the six algorithms. In these plots, the horizontal axis corresponds to the operational time steps, while the vertical axis indicates the well numbers. Figure 7 illustrates the injection control strategy derived from FOA, highlighting considerable fluctuations in injection rates for the same wells across consecutive control intervals. Conversely, RDFOA delivers a more stable and consistent injection plan, as reflected in the smoother curves shown in the figures. Figure 8 compares the liquid production rates across all six methods, clearly demonstrating RDFOA’s superior performance relative to the alternatives.

## Conclusion

In this study, we propose a novel evolutionary algorithm named RDFOA, which enhances the original Fruit Fly Optimization Algorithm (FOA) by integrating a chaotic exploitation mechanism with an orthogonal learning strategy. The chaotic exploitation mechanism improves RDFOA’s capability for global search and escaping local optima, while the orthogonal learning strategy refines the local search process, mitigating the risk of missing the global best solution. To evaluate RDFOA’s effectiveness, extensive experiments were conducted. Ablation studies on the IEEE CEC 2017 benchmark suite confirmed that both mechanisms individually contribute to performance gains. Scalability experiments demonstrated that RDFOA maintains strong stability when addressing large-scale, high-dimensional optimization problems. Additionally, search trajectory analyses illustrate how the population concentrates its search near the global optimum. Comparative testing against various state-of-the-art optimization algorithms further validates the superior performance of RDFOA. Moreover, the practical application of RDFOA in oil and gas production optimization highlights its significant real-world value.

The lack of applying RDDOA to more fields to verify its optimization performance is a limitation of this study. Looking ahead, integrating more complex and advanced mechanisms into FOA is expected to enhance the overall performance of the algorithm and expand its applicability in feature selection, extreme learning machines, and various engineering optimization tasks. Meanwhile, developing a binary version of the algorithm will enhance its ability to solve discrete optimization problems.

## Data Availability

The datasets generated and/or analysed during the current study are available in [https://gitcode.com/open-source-toolkit/0197a/?utm_source=tools_gitcode&index=top&type=card].
